# Forme particulière de Pemphigoide cicatricielle à dépôt unique d'IgA

**DOI:** 10.11604/pamj.2017.26.136.9702

**Published:** 2017-03-13

**Authors:** Amina Aounallah, Mariem Jrad, Mehdi Ksiaa, Sana Mokni, Wafa Saidi, Lobna Boussofara, Badreddine Sriha, Mohamed Denguezli, Najet Ghariani, Colandane Belajouza, Rafia Nouira

**Affiliations:** 1 Université de Sousse, Tunisie; 2 Service de Dermatologie de l’Hôpital Farhat Hached de Sousse, Tunisie; 3 Service de Gastroenterology Hospital Sahloul Sousse, Tunisie; 4 Laboratoire d’Anathomopathlogie de l’Hôpital Farhat Hached Sousse, Tunisie

**Keywords:** Pemphigoide cicatricielle, immunofluorescence directe, sévérité, Cicatricial pemphigoid, direct immunofluorescence, severity

## Abstract

La Pemphigoide cicatricielle est une dermatose bulleuse sous épithéliale qui atteint essentiellement les muqueuses avec une évolution cicatricielle. Il s'agit d'un homme de 66 ans hospitalisé pour gingivite érosive avec dysphagie, dyspnée et flou visuel. L'examen dermatologique retrouvait des lésions érosives du palais et du pharynx. L'examen ophtalmologique notait des symblépharons, un ectropion et une cataracte bilatérale. La biopsie gingivale avait montré un décollement nécrotique de l'épithélium buccal. L'immunofluorescence directe notait un dépôt linéaire d'Immunoglobuline A à la jonction dermo-épidermique. L'Immunofluorescence indirecte était revenue négative. Le diagnostic de pemphigoide cicatricielle était confirmé. Le Transit oeso-gastro-duodénal a objectivé une double sténose de l'œsophage. L'endoscopie nasale, pharyngée et bronchique retrouvait des ulcérations de l'épiglotte, de l'hypopharynx, du pharynx et de l'arbre bronchique. Le patient a bénéficié d'un bolus de Solumedrol relayé par la Prednisone à la dose de 0.5mg/Kg/j associé à la Disulone à la dose de 100mg/j. L'évolution était favorable au début mais s'est compliquée après 2 mois d'une aggravation de la dysphagie et de la sténose oesophagienne. Notre observation est très particulière par la survenue d'une Pemphigoide cicatricielle chez un sujet de sexe masculin ayant un tableau grave en rapport avec l'extension des lésions à toutes les muqueuses conjonctivale, buccale, nasale, œsophagienne et même bronchique associée à une immunofluorescence directe faite d'un dépôt d'IgA uniquement.

## Abstract (en)

Cicatricial Pemphigoid is a subepithelial bullous dermatosis which essentially involves the mucous membranes with cicatricial evolution We report the case of a 66-year old patient hospitalized with erosive gingivitis associated with dysphagia, dyspnea and blurred vision. Dermatologic examination showed erosive lesions involving the palate and the pharynx. Ophthalmologic examination showed symblepharons, ectropion and bilateral cataract. Gingival biopsy revealed a necrotic detachment of the buccal epithelium. Direct immunofluorescence showed linear IgA deposit at the dermo-epidermal junction. Indirect immunofluorescence test was negative. The diagnosis of cicatricial pemphigoid was confirmed. Esophagogastroduodenoscopy objectified double stenosis of the esophagus. Nasopharyngeal and bronchial endoscopy showed ulceration of the epiglottis, hypopharynx, pharynx and bronchial tree. The patient was treated with Solumedrol bolus corresponding to 0.5mg/kg/day prednisone associated with 100mg/day disulone. The patient showed a favorable early clinical outcome complicated because of the aggravation of dysphagia and esophageal stenosis after 2 months. Our case study is singular due to the occurrence of a cicatricial pemphigoid in a male patient with a serious clinical picture due to lesions extending to conjunctival, oral, nasal, esophageal and bronchial mucous membranes associated with direct immunofluorescence only showing IgA deposit.

## Introduction

La pemphigoide cicatricielle est une dermatose bulleuse sous-épithéliale auto-immune rare [1]. Elle se caractérise par une atteinte essentiellement muqueuse notamment la muqueuse buccale, conjonctivale, oropharyngée, oesophagienne [2] avec une évolution cicatricielle des lésions ce qui fait toute la gravité de l'atteinte.

## Méthodes

**Figure 1:**
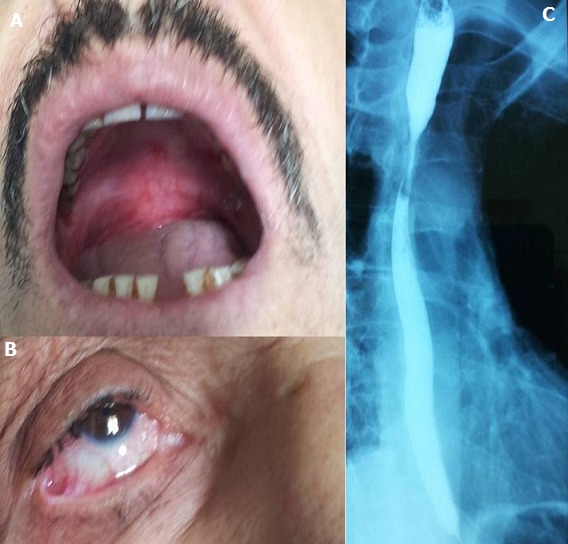
a) multiples érosions au niveau du palais et du pharynx; b) synéchies de la muqueuse conjonctivale Symblepharons and ectropion

**Figure 2:**
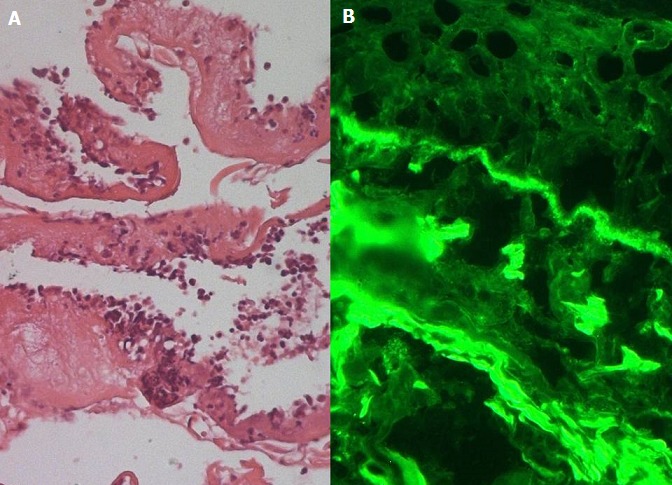
a) décollement nécrotique du revêtement épithélial avec présence d’éléments inflammatoires au niveau de l’interface; b) un dépôt linéaire d’Immunoglobuline A à la jonction dermo-épidermique

Il s'agissait d'un homme âgé de 66 ans, hospitalisé pour une gingivite, une dysphagie haute aux solides, une dyspnée associée à une toux sèche et des lésions buccales associées. Le patient se plaignait de flou visuel depuis 7 mois et de constipation depuis 2 ans. L'examen notait de multiples érosions au niveau du palais et du pharynx (Figure 1 A). L'examen ophtalmologique avait objectivé de multiples symblépharons au niveau du cul de sac inférieur (Figure 1 B), un ectropion des 2 paupières inférieures et une cataracte bilatérale. La biopsie gingivale (Figure 2 A) avait montré un décollement nécrotique de l'épithélium buccal avec présence d'éléments inflammatoires au niveau de l'interface (chorion/épithélium). L'immunofluorescenece directe (IFD) notait un dépôt linéaire d'Immunoglobuline A à la jonction dermo-épidermique (Figure 1 B). L'Immunofluorescence indirecte était revenue négative. Le Transit oeso-gastro-duodénal a retrouvé une double sténose de l'œsophage avec un aspect tubulé de ce dernier. La nasofibroscopie a montré de multiples ulcérations superficielles au niveau de l'épiglotte, l'hypopharynx et du pharynx. L'endoscopie bronchique retrouvait un aspect inflammatoire de tout l'arbre bronchique. L'Exploration fonctionnelle respiratoire notait un trouble ventilatoire restrictif léger. La biologie avait retrouvé une anémie normochrome normocytaire associée a une hyperalpha 1 globulinémie, hyperalpha2 globulinémie et une hypergammaglobulinémie. Le patient a bénéficié d'un bolus de Solumedrol 3 jours de suites relayé par Prednisone à la dose de 0.5mg/Kg/j associé à la Disulone à la dose de 100mg/j. L'évolution était favorable au début mais s'est compliquée au bout de 2 mois d'une aggravation de la dysphagie et de la sténose oesophagienne nécessitant des dilatations bronchiques et une augmentation de la dose de la corticothérapie générale.

## Discussion

La pemphigoide cicatricielle est une dermatose bulleuse auto-immune sous épidermique rare [1]. Elle est plus fréquente chez la femme (sexe ratio F/H de 1.5 à 2) [3]. La découverte de la maladie se fait suite aux symptômes buccaux dans 80 à 90% des cas comme c'était le cas chez notre patient. Différentes localisations des lésions muqueuses peuvent se voir [3]. L'atteinte buccale et oculaire constituent les localisations les plus fréquentes [3]. L'étendue des lésions muqueuses conditionnent la gravité de la maladie ainsi que leur chronicité [4]. Notre observation est originale par la survenue de la pemphigoide cicatricielle chez un patient de sexe masculin avec une extension des lésions à la muqueuse buccale, oculaire, pharyngolaryngée, œsophagienne et même trachéobronchique. En effet, l'atteinte de l'œsophage est rare, retrouvée dans 5 à 15% des cas [3]. La sténose œsophagienne est constatée dans 4% des cas seulement. L'inflammation bronchique retrouvée chez notre patient n'a pas été décrite auparavant. L'histologie typique au cours de la pemphigoide cicatricielle objective une bulle sous épidermique, sans acantholyse, ni nécrose du toit et est donc indifférenciable de celle de la pemphigoïde bulleuse. Le plancher de la bulle est le siège d'un infiltrat de polynucléaires neutrophiles et/ou éosinophiles. L'IFD, retrouve un dépôt linéaire, continu d'IgG et/ou de C3 le long de la membrane basale, souvent associés à des IgA [5]. Certains auteurs suggèrent que les patients présentant à l'IFD un dépôt d'IgA associé aux IgG présenteraient un tableau plus grave et persistant [4]. Ceci était constaté chez notre patient vu qu'il a présenté un dépôt d'IgA isolés. La détection par immunofluorescence indirecte (IFI) des anticorps de type IgG et IgA dirigés contre la membrane basale est un facteur prédictif de la sévérité de la maladie [3]. Chez notre patient l'IFI était négative. L'immunoblot révèle une positivité pour la 180-KD bullous pemphigoid antigen (BPAg2), les sous unités α6/β4 de l'intégrine ainsi que l'épiligrine et le collagène de type VII [3]. Sur le plan thérapeutique, on préconise d'associer une corticothérapie générale à la dose de 1mg/kg/j à un traitement par Dapsone à la dose de 50 à 200mg/j plus ou moins associées aux immunosuppresseurs type Azathioprine (100-150 mg/J) ou Mycophenolate Mofetil (1-1.5 g/J) [6]. D'autres études ont proposé les anti-tumor necrosis factor (TNF)αet les immunoglobulines en IV chez les patients réfractaires aux thérapeutiques précédentes [7].

## Conclusion

Notre observation est très particulière par la survenue d'une Pemphigoide Cicatricielle chez un sujet de sexe masculin ayant un tableau grave en rapport avec l'extension des lésions à toutes les muqueuses conjonctivale, buccale, nasale, œsophagienne et même bronchique associée à une IFD faite d'un dépôt d'IgA uniquement. Cette observation illustre bien la gravité du tableau clinique et les difficultés thérapeutiques associées des patients présentant une Pemphigoide Cicatricielle et qui ont à l'IFD un dépôt d'IgA linéaire. Ces constatations devraient être appuyées par des études à grand effectif multicentriques et internationales afin de mieux étudier cette corrélation anatomoclinique.

### Etat des connaissances actuelles sur le sujet

- La pemphigoide cicatricielle est une dermatose bulleuse autoimmune de la jonction dermo-épidermique et touche les muqueuses;
- Les facteurs prédictifs de l'évolution et du pronostic de la pemphigoide cicatricielle sont peu étudiés.

### Contribution de notre étude à la connaissance

- Le dépôt d'IgA unique au cours de la pemphigoide cicatricielle serait associé à une forme étendue et grave de cette dermatose. Il s'agit d'une constatation qui devrait être confirmée par des études à grande échelle et multicentrique.
